# Duodenum Inversum: A Rare Cause of Chronic Nausea and Vomiting

**DOI:** 10.1155/2018/7538601

**Published:** 2018-12-25

**Authors:** Saurabh Chandan, Ojasvini Choudhry Chandan, Alexander Todd Hewlett

**Affiliations:** ^1^Department of Gastroenterology and Hepatology, University of Nebraska Medical Center, Omaha NE, USA; ^2^Department of Pediatric Gastroenterology, Hepatology and Nutrition, University of Nebraska Medical Center, Omaha NE, USA

## Abstract

Duodenum inversum (DI), also known as inverted duodenum or duodenum reflexum, is a congenital malformation in which the third portion of the duodenum, instead of continuing leftward to the ligament of Treitz, reverses direction and travels in a superior, posterior track prior to crossing the midline above the pancreas. We present a case of a 62-year-old woman presenting with chronic nausea and vomiting, subsequently found to have DI.

## 1. Introduction

Duodenum inversum (DI) was first described in 1940 by Feldman and Morrison who described 14 such cases in 20,000 gastrointestinal X-ray examinations, with an incidence of 0.07 per cent [1]. Since 1950, only 18 cases have been reported in literature with the majority being in adult population. This condition can be difficult to diagnose and is often confused with other, more common, anomalies of intestinal rotation such as malrotation, incomplete rotation, annular pancreas, and pancreas divisum [2–4].

## 2. Case Presentation

A 62-year-old female with a medical history of generalized anxiety disorder and hyperlipidemia presented to our clinic for further evaluation of chronic nausea and chronic intermittent abdominal pain ongoing for over 10 years. She also reported occasional nonbloody, nonbilious emesis along with the nausea which was not exacerbated by oral intake. Her symptoms were refractory to oral Ondansetron, Metoclopramide, and Promethazine. She denied bloating, weight loss, or changes in bowel habits. Her past surgical history only included an uncomplicated laparoscopic cholecystectomy.

On physical exam her abdomen was soft and nontender with normoactive bowel sounds. Laboratory study results showed a hemoglobin level of 12.2 g/ml (normal 11-15.1 g/dl), total bilirubin level of 0.8 mg/dL (normal 0.3-1 mg/dl), alkaline phosphatase of 74 U/L (normal 32-91 U/L), aspartate aminotransferase (AST), and alanine aminotransferase (ALT) levels of 32 and 41 U/L, respectively (normal 15-41 U/L, 7-52 U/L). A random cortisol level was 12 mcg/dl.

**Figure 1:**
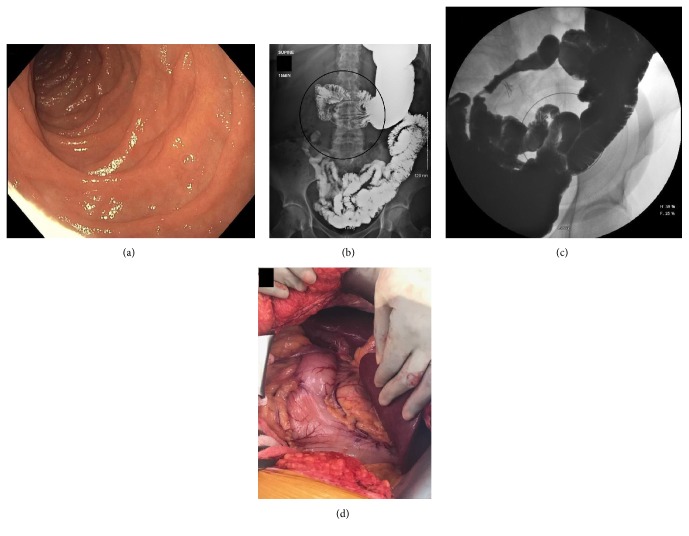
(a) Endoscopic image, normal duodenum. (b)-(c) Upper gastrointestinal series with small bowel following through with barium contrast. (d) Exploratory laparotomy.

She was initially sent for a CT angiogram of the abdomen with intravenous contrast which did not show any radiographic evidence of median arcuate syndrome. She then underwent a diagnostic esophagogastroduodenoscopy which revealed a normal duodenum (Figure 1(a)). An upper gastrointestinal series with small bowel follow through using barium contrast showed no evidence of gastric outlet obstruction; however, the duodenal course was abnormal, with the proximal portion looping back on itself in the right abdomen and extending superiorly to the level of the duodenal bulb (Figure 1(b)) before crossing the midline with loops of small bowel in the left upper quadrant (Figure 1(c)). Based on these characteristic radiographic findings, the diagnosis of duodenum inversum was made and the patient was referred for possible surgical management. She underwent an exploratory laparotomy which showed proximal loops of jejunum adhered to the right lower quadrant and patulous appearing first and second portions of the duodenum (Figure 1(d)). An end-to-side duodenojejunostomy was then performed successfully. The patient had no procedure related complications and began tolerating oral intake at postoperative day 4. She was subsequently discharged home in good condition and remained symptom-free at follow-up.

## 3. Discussion

Duodenum inversum is thought to develop due to persistence of the dorsal mesentery with a mobile duodenum [5, 6]. Other congenital anomalies in fixation or position of the right kidney, pancreas, and transverse mesocolon are commonly associated with this condition. Duodenum inversum may mimic superior mesenteric artery syndrome and must be differentiated from redundancy of the first part of the duodenum, malrotation, closed duodenal loops and left-sided duodenum of situs inversus [7]. There are 4 subtypes of duodenum inversum described in literature, but this classification has limited value [8]. Although it can occur at any age, the average age of diagnosing DI is 46 years with a male-to-female ratio of 4:1. The diagnosis is primarily made by radiological evaluation ordered for patients with chronic abdominal pain [4, 9]. The classic findings on an upper gastrointestinal series with barium contrast show (1) return of the contrast substance from the second into the first part of the duodenum and then into the bulb more frequently; (2)* stasis *in the duodenum; and (3) rapid passage of the contrast medium through the third stage. While not associated with significant morbidity or mortality, DI remains an important diagnostic consideration as it can present with proximal gastrointestinal obstruction [10].

Medical management includes acid suppression due to risk of duodenitis and remains the mainstay of treatment for patients without complications such as bowel obstruction [4, 6]. Failure to improve with medical therapy may be an indication for surgical intervention. While several procedures have been successfully tried in the past [4, 5, 7], currently, there is no gold standard for treatment. Our patient was successfully treated with an end-to-side duodenojejunostomy.
